# Resilience of *Stevia rebaudiana* (Bertoni) Bertoni in the Underwater Biospheres of Nemo’s Garden^®^: Adaptation to New Cultivation Systems

**DOI:** 10.3390/molecules27238602

**Published:** 2022-12-06

**Authors:** Roberta Ascrizzi, Marinella De Leo, Laura Pistelli, Claudia Giuliani, Ylenia Pieracci, Barbara Ruffoni, Carlo Mascarello, Gelsomina Fico, Guido Flamini, Luisa Pistelli

**Affiliations:** 1 Dipartimento di Farmacia, Università di Pisa, Via Bonanno Pisano 6, 56126 Pisa, Italy; 2 Centro Interdipartimentale di Ricerca “Nutraceutica e Alimentazione per la Salute” (NUTRAFOOD), Università di Pisa, Via del Borghetto 80, 56124 Pisa, Italy; 3 Dipartimento Scienze Agrarie, Alimentari e Agro-Ambientali, Università di Pisa, Via del Borghetto 80, 56124 Pisa, Italy; 4 Dipartimento di Scienze Farmaceutiche, Università di Milano, Via Mangiagalli 25, 20133 Milan, Italy; 5 Orto Botanico G.E. Ghirardi, Dipartimento di Scienze Farmaceutiche, Università di Milano, Via Religione 25, 25088 Toscolano Maderno, Italy; 6 CREA-Centro di Ricerca Orticoltura e Florovivaismo, Corso Inglesi 508, 18038 Sanremo, Italy

**Keywords:** sustainability, steviol glycosides, polyphenols, LC-MS, β-caryophyllene, GC-MS, volatiles, head space, climate change

## Abstract

The Nemo’s Garden^®^ project is an alternative production system for areas with scarce cultivable land but significant presence of water; thus, it is an interesting intervention to address the climate crisis. This work aimed to evaluate the micromorphological, biochemical, and phytochemical characteristics of *Stevia rebaudiana* (Bertoni) Bertoni grown underwater compared to the terrestrial specimens. The micromorphological analyses, performed on the leaves using light microscopy, fluorescence microscopy, and scanning electron microscopy, evidenced a general uniformity of the trichome morphotype and distribution pattern. The histochemical investigation indicated the simultaneous presence of terpenes and polyphenols in the trichome secreted material from the underwater samples and a prevailing polyphenolic content in the terrestrial specimens; this was also confirmed by biochemical analyses (26.6 mg GAE/g DW). The characterization of non-volatile components, performed using HPLC–MS, showed similar chemical profiles in all the samples, which were characterized by phenolic compounds and steviol glycosides. The volatile compounds, evaluated using HS-SPME coupled with GC–MS, showed sesquiterpene hydrocarbons as the main class in all the analyzed samples (80.1–93.9%). However, the control plants were characterized by a higher content of monoterpene hydrocarbons (12.1%). The underwater biosphere environment did not alter *S. rebaudiana* micro-morphological characters, although slight qualitative changes were evidenced for the compounds produced as a response to the growth conditions.

## 1. Introduction

The climate crisis is the toughest challenge the world is facing today, as reported by the Intergovernmental Panel on Climate Change (IPCC) [1]. The United Nations and Governments are recognizing the urgency of a quick, combined intervention on multiple levels, as emerged in the 2021 Pre-COP26 held in Milan, Italy and then in the 2021 COP26 presided in Glasgow, Scotland [2]. Among the possible interventions, a redesign of the agricultural models must be considered as the unsustainability of the traditional methods is now evident. Despite being one of the major causes of climate change, agriculture also suffers the consequences of this dramatic situation because of rising temperatures, variability in precipitation, scarcity of water, and many other unfavorable conditions [3], which are expected to affect crop productivity all over the world [4]. To make matters worse, a world population growth of about two billion is estimated by 2050, which necessarily entails a greater pressure on the agricultural system to produce 80% more food globally; this will have a consequential depletion of the soils already cultivated, due to the lack of available lands that can be employed without compromising the ecosystem biodiversity [3,5]. It is evident that these global trends need concrete measures to reduce the impact of agricultural production, leading to a sustainability transition [6].

The Nemo’s Garden^®^ Project (Genova, Italy) may be an interesting alternative production system to address both the scarcity of cultivable lands and climate change, especially in areas where the presence of water presence significant (e.g., islands, lakes, and flooded areas). At present, the Nemo’s Garden^®^ consists of six underwater greenhouses, called “biospheres”, located in Noli’s Bay, close to Savona (Italy). 

This innovative pilot underwater farm has been developed to help agricultural development, in particular in those areas characterized by difficult environmental, economic, and geo-morphological conditions [4]. Despite the fact that the project was conceived in 2012, the cultivation inside the greenhouses started in 2015 with in-ground cultivation, which was followed by the development of the hydroponic system some years later [7]. 

*Stevia rebaudiana* (Bertoni) Bertoni was one of the plant species selected for underwater growth in the Nemo’s Garden^®^ biospheres [7]. It is a perennial herbaceous plant belonging to the Asteraceae family [8]. *S. rebaudiana* is an obligate short-day plant, so long-day prolongs the vegetative phase and increases its growth [9]. In recent times, it has gained increasing attention for its ability to biosynthesize steviol glycosides, which are molecules with a high sweetening power [10,11]. Steviol glycosides are a class of secondary metabolites all characterized by the same tetracyclic diterpenoid aglycone, steviol, but differ in the carbohydrate residues [12]. More than 60 compounds belonging to this class can be found in the plant leaves, including stevioside, rebaudioside (A to F), steviolbioside, and isosteviol; however, stevioside, rebaudioside A, and rebaudioside C are the main ones [13]. Although steviol glycosides are considered the most promising bioactive compounds, they are not the only phytochemicals produced by the plant. The leaves of *S. rebaudiana* are an interesting source of other molecules with pharmacological and health-promoting properties, such as polyphenols and terpenoids, as well as phytosterols and vitamins [11,14]. 

This complex phytochemical profile makes *S. rebaudiana* an interesting plant that is exploitable in different fields of application; in addition, its ability to successfully grow in a wide variety of habitats makes it even more attractive [12,14]. *S. rebaudiana*, indeed, can be grown from semi-humid, subtropical to temperate environments as a multi-annual or annual crop, respectively [14]. Despite its extreme adaptability, environmental conditions are able to affect plant growth and development, as well as its productivity in terms of secondary metabolites [10]. 

Following our previous report on basil plants grown inside the biospheres [4], the present work deals with the evaluation of the effect of the Nemo’s Garden^®^ underwater environment on the different features of *S. rebaudiana*. The performed analyses investigated the (i) micromorphological, (ii) biochemical, and (iii) phytochemical characteristics of plants grown underwater (in pot and in a hydroponic system) compared to a specimen grown in a terrestrial greenhouse (in pot). 

## 2. Results

### 2.1. Micromorphological Analyses

**Figure 1:**
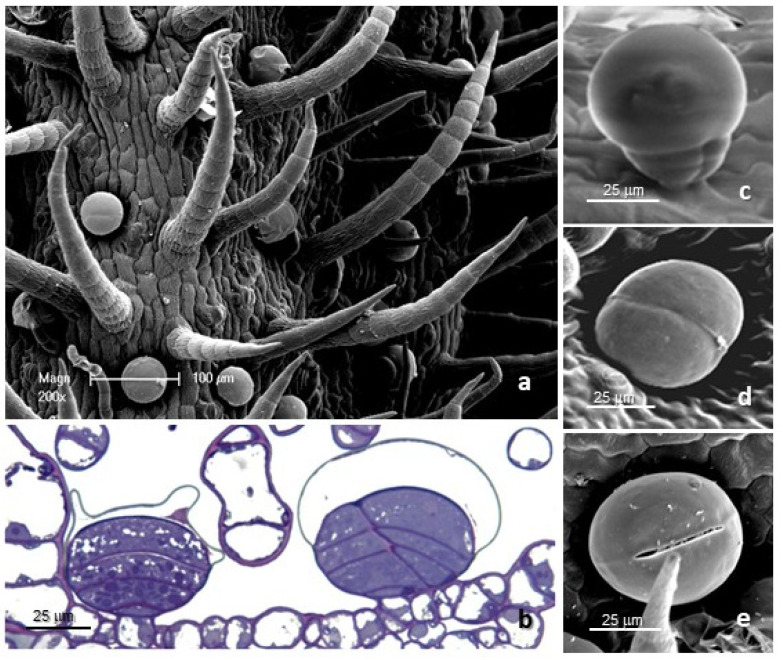
SEM and LM micrographs showing the covering and glandular trichomes observed on the leaves of the examined specimens of *Stevia rebaudiana*. (**a**) Detail of the abaxial leaf surface with covering uniseriate trichomes and biseriate glandular hairs. (**b**) Longitudinal section of biseriate 10-celled glandular trichomes stained with Toluidine Blue. (**c**) Protruding biseriate 10-celled glandular trichome. (**d**) Sunken biseriate glandular trichome with intact cuticle. (**e**) Sunken glandular trichome showing the rupture of the cuticle along a predermined line of weakness.

**Figure 2:**
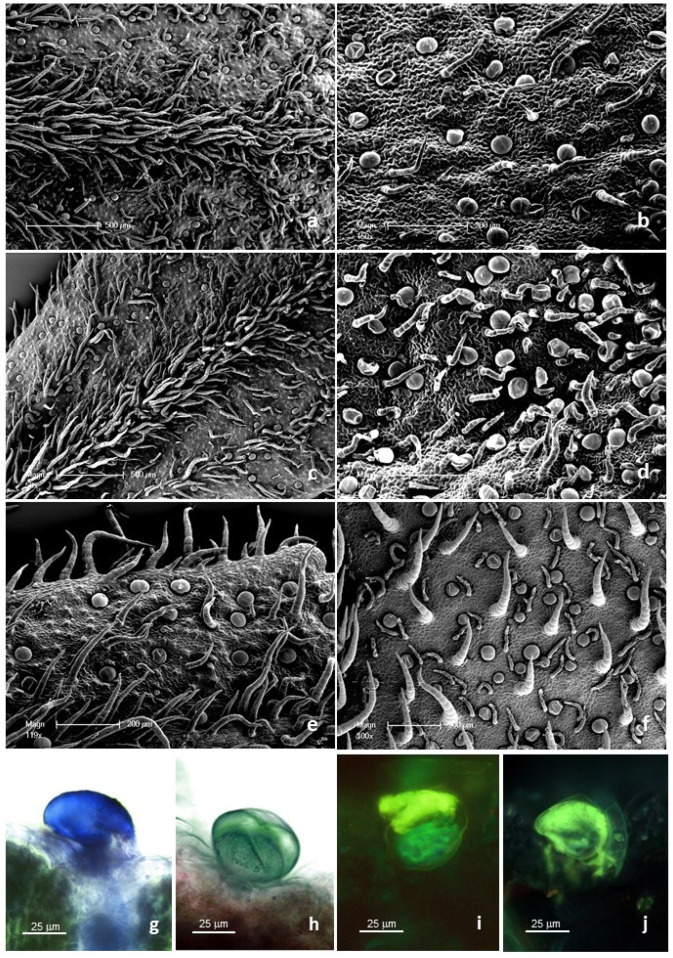
(**a**–**f**) SEM micrographs showing the distribution pattern of the covering and glandular trichomes on the leaves of the examined specimens of *Stevia rebaudiana*. (**a**,**b**) Details of the abaxial (**a**) and adaxial (**b**) leaf surfaces of the underwater-grown samples (hydroponic). (**c**,**d**) Details of the abaxial (**c**) and adaxial (**d**) leaf surfaces of the underwater-grown samples (pot). (**e**,**f**) Details of the abaxial (**e**) and adaxial (**f**) leaf surfaces of the terrestrial samples (pot). (**g**–**j**) LM and FM micrographs showing the histochemistry of the glandular trichomes on the leaves of the examined specimens of *Stevia rebaudiana*. (**g**) Nadi reagent. (**h**) Ferric Trichloride. (**i**) Naturstoff reagent A. (**j**) Aluminum Trichloride.

The leaf *indumentum* of the underwater-grown samples and the terrestrial specimens exhibited a general consistency both in terms of trichome morphotypes and distribution pattern in all the replicates (Figure 1a and Figure 2). Both non-glandular and glandular hairs were observed (Figure 1a–e). The former were simple, multicellular, and uniseriate, with 5–7 cells of progressively smaller diameter from the base towards the apex (Figure 1a); the basal cells were usually protruding from the leaf epidermis and sporadically appeared swollen. They were located on both leaf surfaces (Figure 2a–f), being mainly arranged at the median rib on the abaxial side and uniformly distributed on the lamina of the adaxial side. The glandular trichomes belonged to a single morphotype (Figure 1b–e): 10-celled and biseriate, composed of two rows of five cells each (two basal cells, two stalk cells, and six secretory cells). The apical secretory cells were surmounted by a large and prominent subcuticular space, formed by the detachment of the cuticle that lined the apical secretory cells (Figure 1), so that the mature glands presented a typical spherical shape (Figure 1b). These hairs were generally sunken or might slightly protrude from the epidermis (Figure 1c,d) as a consequence of the different elongation of the basal cells. 

The secretion was released externally following the rupture of the cuticle, along a predetermined line of weakness on the apical cuticular layer of the mature glands (Figure 1e), and the secretion products of the biseriate glandular trichomes exhibited positive responses to both lipophilic tests, including Fluoral Yellow-088 and Nadi reagent (Table 1, Figure 2g), to the FeCl_3_ test (Figure 2h), specific to polyphenols, and to the AlCl_3_ and Naturstoff reagent A tests (Figure 2i,j), specific to flavonoids and flavonols, respectively. They showed weak coloration or negative responses to the hydrophilic dyes Alcian Blue and Ruthenium Red. 

The histochemical investigation indicated the simultaneous presence of comparable fractions of terpenes and polyphenols in the trichome secreted material from both underwater-grown samples, whereas a prevailing polyphenolic content was found in the trichomes from the terrestrial specimens. Concerning the mesophyll tissues, the histochemical observations turned out to be uneven, since the results were generally negative for all the employed dyes, with sporadic weakly positive responses only for polyphenols.

### 2.2. Biochemical Analyses

The content of chlorophylls was first examined to highlight any plant resilience to the underwater cultivation (Table 1). The total chlorophyll content did not differ between the control sample and the Nemo’s sample grown in hydroponic solution, showing 1.09 and 1.05 mg/g FW, respectively. The plants cultivated in pot in the biosphere, instead, showed a lower content of chlorophylls (0.81 mg/g FW), mainly due to the lower chlorophyll a content, while the amount of chlorophyll b was similar to the other samples. 

The different methods of cultivation resulted in a different content of some metabolites in the analysed plants of *S. rebaudiana*. The highest carotenoid content was found in the hydroponic sample, while the plants grown in pot in the Nemo’s Garden^®^ showed very low amounts of carotenoids. The control plants, instead, presented an intermediate level of these metabolites. 

Other compounds, such as total polyphenols, also showed different values: the control plants contained 26.6 mg/g DW, approximately two fold the content of the plants cultivated underwater, which accounted for up 15.83 and 10.55 mg/g DW for the hydroponic and potted plants, respectively. 

Analogous to the polyphenol content, the antioxidant activity, as determined by both DPPH-assay and ABTS assays, showed the same trend for polyphenols: it was higher for the control plants grown in pot in terrestrial conditions than in the plants cultivated in the underwater biospheres. However, the antioxidant power of the hydroponic sample was slightly greater than that of the Nemo’s potted sample.

### 2.3. Phytochemical Analyses

#### 2.3.1. Non-Volatile Compound Analysis

##### Chemical Characterization

**Figure 3:**
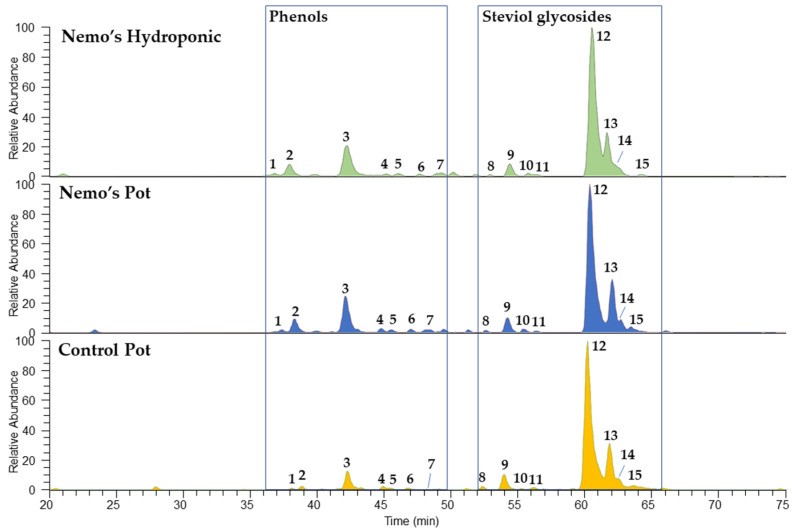
Chemical profiles of non-volatiles detected in *Stevia rebaudiana* methanolic extracts using HPLC-ESI-MS registered in negative ionization mode. The peak numbers correspond to those of Table 2.

All methanolic extracts of *S. rebaudiana* were subjected to HPLC-PDA-ESI-MS/MS analyses in order to characterize the non-volatile components and to compare the chemical profile of the three samples. The obtained chromatograms are shown in Figure 3.

Compared to the control, both the Nemo’s Garden^®^ samples showed superimposable chemical profiles, all characterized by the presence of phenols and steviol glycosides. The tentative identification of all molecules was performed based on the full and fragmentation mass data and their comparison with the literature data (Table 3). The MS/MS experiments were performed in negative ionization mode for phenols, while the positive ionization mode was more useful for the fragmentation of steviol glycosides. Furthermore, the maximum absorbance was registered for phenols, whereas no UV data were obtained for steviol glycosides. According to the literature [15,16,17], the phenolic fraction was composed of a number of dicaffeoylquinic acid isomers (compounds **1**–**4**), as deduced from the deprotonated molecular ions [M-H]^−^ at *m*/*z* 515 and the generated product ions at *m*/*z* 353 and 179 corresponding to the residues of chlorogenic and caffeic acids, respectively. Together with hydroxycinnamic acids, two quercetin glycosides were detected, assigned as quercetin rhamnoside (compound **4**) and quercetin rhamnosyl-diglucoside (compound **6**), as deduced from the loss of hexose (-162 u) and deoxyhexose (-146 u) units from the quercetin aglycone (product ion at *m*/*z* 301). All compounds showed two characteristic maximum absorbances at 248–257 and 328–335 nm, which are typical of these classes of metabolites. The steviol glycoside fraction was a mixture of eight different molecules (compounds **8**–**13**), where **12** and **13** were the most representative ones. All compounds were detected in both positive and negative ionization modes, but, in our experiments, the more informative data were obtained from the analysis of the fragmentation pathways of sodiated molecular adducts [M+Na]^+^. When the parent ion was subjected to a collisional energy, the loss of hexose or deoxyhexose units was observed. The detected compounds showed similar fragmentation patterns due to their structural similarity, except for compounds **11** and **12**, whose fragmentation spectra showed very low intensity, probably due to their occurrence only in traces in the analyzed extracts. Based on the comparison of MS data and elution order, the sweet diterpenoid glycosides were tentatively identified as rebaudioside A (**8**), rebaudioside D (**9**), rebaudioside N (**10**), rebaudioside F (**11**), stevioside (**12**), rebaudioside C (**13**), and rebaudioside B (**14**), according to the literature data [18,19,20,21].

##### Quantitative Estimation of Steviol Glycosides

The amount of the major steviol glycosides **12** (stevioside) and **13** (rebaudioside C) in all *S. rebaudiana* extracts was established by constructing a calibration curve using stevioside as a reference standard. As deduced from the results shown in Table 3, the content of stevioside was comparable in both the control and Nemo’s hydroponic plants, while a decrease was observed in the underwater potted sample. The content of rebaudioside C was similar in the control and Nemo’s potted samples, while it was almost doubled in the Nemo’s hydroponic sample.

#### 2.3.2. Volatile Compound Analysis

The composition of the headspace (HS) of the three samples is reported in Table 4. Sesquiterpene hydrocarbons (SHs) prevailed in all compositions, as they accounted for over 90% in both biosphere samples and for about 80% in the control sample. For this class, the differences between the two growing sites were mainly quantitative, rather than qualitative. Among SHs, β-caryophyllene was detected as the most abundant compound in all the samples, and, in the HS of the control sample, it reached up to 36.0%. Both biosphere samples showed qualitatively and quantitatively comparable HS compositions, with β-caryophyllene (27.5 and 21.1% in pot and hydroponic sample, respectively), followed by bicyclogermacrene (14.8 and 19.5% in the potted sample and the hydroponic sample, respectively), (*E*)-β-farnesene (10.0 and 10.4%), and germacrene D (10.9 and 10.9%) as main components.

The main qualitative difference between the biosphere samples and the control was evidenced for the presence of monoterpene hydrocarbons (MHs). While this class only accounted for 1.3 and 1.9% in the biosphere potted sample and the hydroponic sample, respectively, they were detected in relative abundance, accounting for up to 12.1%, in the control sample, of which 9.1% was represented by β-pinene.

The other detected chemical classes in the analyzed plants accounted for less than 3.8% in all samples. Oxygenated sesquiterpenes (OSs) were slightly more abundant in the hydroponic sample (2.7%), while they represented 2.1 and 2.2% in the biosphere potted sample and in the control sample, respectively. For the biosphere samples, however, the most abundant OSs were germacrene-D-4-ol and caryophyllene oxide; for the control sample, it was (*E*)-nerolidol, followed by germacrene-D-4-ol. Oxygenated diterpenes (ODs) were only represented by manoyl oxide in all the HSs, with a slightly higher relative presence in the control sample (3.8%) compared to the biosphere ones (0.9% and 1.8% in the potted and hydroponic samples, respectively).

## 3. Discussion

Across the world, many areas are subjected to flooding, with catastrophic consequences on field crops. To contribute to the fight against climate change, in the present study, the effect of the Nemo’s Garden^®^ underwater environment on the different features of *S. rebaudiana* was evaluated. The goal was to encourage new alternative production systems that are able to address the availability of cultivable lands. The starting point of the work was a comparison between the plants cultivated in the underwater system and the terrestrial plants. However, as the experiment progressed, an underwater hydroponic system was implemented because it is known that hydroponic cultivation is preferred to reduce the proliferation of soil mould due to high humidity, which is present in the biosphere. 

The combined use of SEM and histochemical dyes allowed us to extensively describe the morpho-functional features of trichomes, integrating the available literature data [22,23,24]. Similar to most species of the family Asteraceae, the *indumentum* of the epigeal parts of *S. rebaudiana* was characterized by both non-glandular and glandular trichomes. Their morphological features and distribution pattern were consistent with previous reports [22,23,24]. With special emphasis on the glandular trichomes, they appeared generally sunken on the leaf epidermis, as already described by Tateo et al. [23]. The secretory materials were entrapped between the cuticular sheath and the head calls, being released by the tearing or rupture of the cuticle [25]. The SEM observations indicated that, in *S. rebaudiana,* the cuticular sheath ruptured along a predetermined line running along the middle of the gland head, as already reported [23]. However, cuticular exudation might also occur, as stated by other authors [26]. The histochemical tests revealed that the material secreted mainly consisted of phenols and terpenes. Tateo et al. also reported alkaloid production [23]. 

The investigated samples exhibited comparable overall micro-morphological features, with the exception of the prevailing polyphenolic content in the glandular trichomes from the terrestrial specimens. However, the analyzed plants appeared well adapted to develop in all the analyzed growth conditions. The same result was also obtained from the biochemical analysis, which highlighted the lower content of polyphenols in the Nemo’s plants than in the control plants and, as a consequence, their lower antioxidant activity, since it is well known that polyphenols work as scavengers of free radicals and as natural metal chelators [27]. This discrepancy between the plants cultivated in the biosphere environment and in the terrestrial conditions was attributable to the different environments of cultivation responsible for the different size and behavior of the plants. Previous research has clearly demonstrated the influence of nutrient fertilization, harvest time, and other stressors (as drought) on the production of metabolites, such as polyphenols and steviol glycosides [28]. Moreover, another determining factor that influences metabolite production should be light intensity; recently, it has been proven by some authors [9] that *S. rebaudiana* growth is affected by light intensity, which could also contribute to the changes in metabolic responses. Jarma-Orozco et al. showed that plants grown in bio-spaces, with greenhouse-based technologies and reduced UV radiation, showed a better photosynthetic performance. In this respect, the content of chlorophyll a is usually higher than that of chlorophyll b, resulting in a chl a/chl b ratio greater than zero, and a high ratio should be considered a symptom of both good plant cultivation and optimal photosynthetic apparatus function [29]. In the present work, the total chlorophyll content, driven by both chlorophyll a and b amounts, was evaluated. The results highlighted that the hydroponic plants growth in the Nemo’s Garden^®^ showed a similar content of these pigments to the control plants, while the plants cultivated in the in-ground system of the biosphere was characterized by lower amounts. Therefore, the value of chl a/chl b ratio demonstrated that the plants grown in pot underwater were not in an optimal condition; this was in contrast to both the control and Nemo’s hydroponic plants, which showed a higher ratio value. 

Furthermore, the light regime is considered able to influence steviol glycosides production. However, the optimal light regime to improve their content is currently under debate [30]. As evidence for this aspect, in our work, from a qualitative point of view, the growth conditions in the underwater biospheres did not alter the chemical profile in terms of non-volatile secondary metabolites. Indeed, the three analyzed samples showed similar metabolomic profiles, with 14 major compounds tentatively identified by LC–MS techniques as phenolic compounds and steviol glycosides; this was in agreement with previous data reporting about the chemical composition of *S. rebaudiana*. Stevioside and rebaudioside C were the most representative steviol glycosides in the three samples, but the quantitative analysis highlighted a small variation in their content. The Nemo’s potted sample showed the lowest content of stevioside (Table 4), as well as a lower extraction yield (Table 1). The total amount of steviol glycosides can be subjected to large differences due to environmental, agronomic, geographic, and genetic factors [20]. Thus, the biosphere growth conditions might have affected the metabolic activity, even though the quality of the grown plants remained good, since both phenols and sweet diterpenoid glycosides were produced. The effect of the underwater conditions was also evidenced for the volatile spontaneous emissions. All the analyzed samples were characterized by sesquiterpenes hydrocarbons being the most represented chemical class of compounds, in accordance with Tateo et al. [23]. However, as previously reported, the main qualitative difference between the biosphere samples and the control was a markedly lower content of monoterpene hydrocarbons in the Nemo’s Garden^®^ plants, while β-pinene represented the main component of the control sample’s volatile emission. It was interesting that the relative amount of β-pinene was markedly lower in the Nemo’s pot than in the terrestrial pot, but very similar to that of the hydroponic plant grown in the biosphere. Conversely, sesquiterpenes bicyclogermacrene and δ-elemene were detected in higher amounts in both Nemo’s samples than in the control sample. As reported by Benelli et al. [31], the larger emission of both of these compounds were related to the plants being less attractive to pollinators. 

## 4. Materials and Methods

### 4.1. Chemicals

Methanol and formic acid for HPLC analyses were purchased from VWR (Milan, Italy). HPLC grade water (18 mΩ) was obtained using a Mill-Ω purification system (Millipore Corp., Bedford, MA, USA). Stevioside standard was purchased from Merck (Darmstadt, Germany). 

### 4.2. Plant Material 

#### 4.2.1. Control Potted Plants

*Stevia rebaudiana* (Bertoni) Bertoni control plants were cultivated in pots in a greenhouse of CREA-Centro di Ricerca Orticoltura e Florovivaismo (Sanremo-Italy) according to the method reported by Sacco et al. [32]. *Stevia* cuttings (8 cm) from mother plants cultivated in the greenhouse were grown in pots with commercial soil for sowing and perlite for 30 days to obtain a good rooting. The rooted plants were repotted in pots of 14 cm in diameter that were kept in a greenhouse and contained a commercial substrate consisting of a mixture of blonde and brown peat drained with 30% of pumice of different granulometry, and a slow-release fertilizer was added that lasted 2 months. The collection was performed at the same phenological stage as the biosphere samples. Inside the greenhouse, the maximum daily light intensity ranged between 23,000 and 35,000 lx (605–920 μmol/m^2^/s), and the daily temperature was between 18.0 and 30.0 °C (with a mean ΔT of 7.0 °C). The mean daily relative humidity ranged between 42 and 63%, depending on the day. 

#### 4.2.2. Plants from Nemo’s Garden^®^ Biosphere

Some of the rooted plants (30 days old) cultivated by CREA-Centro di Ricerca Orticoltura e Florovivaismo (Sanremo-Italy) were transferred in July–August 2019 into a Nemo’s Garden^®^ biosphere (GPS coordinates: 44.2011960445432, 8.418202272644171), located 5 m below the sea level, and were maintained in soil or transferred into the hydroponic system, developed as described in Dini et al. [7], for two months. For both the pots and the hydroponic system, a fertilizing solution (5% *v*/*v* of Aerogarden) was provided every 2 weeks. Inside the biosphere, only the natural light was exploited: the maximum light intensity ranged between 152–190 μmol/m^2^/s with natural photoperiod. The daily temperature ranged between 27 and 30 °C. The temperature variation between day and night was around 3–4 °C, with an average relative humidity around 80%. The samples were collected and used either fresh or dried under natural room conditions. To avoid burning damages, the samples were kept away from direct light prior to analyses.

### 4.3. Micromorphological Analyses

Fresh mature leaves for micromorphological investigation were gathered concurrently to the collection of plant material for both biochemical and phytochemical analyses. The collection was performed for both the underwater-grown samples and the terrestrial specimens.

At least ten leaves, similar in total size, position, and developmental stage, were selected from all the specimens to assess the level of variability in trichome morphotypes, distribution pattern, and histochemistry of the secreted material. Light microscopy (LM), fluorescence microscopy (FM), and scanning electron microscopy (SEM) were used.

#### 4.3.1. LM and FM

The fresh samples were frozen, sectioned, and stained with various histochemical techniques to evidence the chemical nature of the secretory products and to specifically locate the sites of synthesis, storage, and release of the secondary metabolites. The following methods were employed: Fluoral Yellow 088 for total lipids; Nile Red for neutral lipids; Nadi reagent for terpene; Ruthenium Red and Alcian Blue for acidic polysaccharides; Ferric Trichloride for polyphenols; Aluminum Trichloride for flavonoids; and Naturstoff reagent A for flavonols. The control procedures were performed simultaneously. Observations were made with a Leitz DM-RB Fluo optical microscope.

#### 4.3.2. SEM

The plant samples were hand-prepared, fixed in a F.A.A. solution (Formaldehyde:Acetic Acid:Ethanol 70% = 5:5:90) for 24 h at 4 °C, dehydrated in an ascending ethanol series up to absolute, and then dried using a critical-point-dryer apparatus. The samples, mounted on aluminum stubs, were coated with gold and observed with a Philips XL 20 SEM operating at 10 kV.

### 4.4. Biochemical Analyses

#### 4.4.1. Pigments and Polyphenols, Extraction and Determination

Chlorophyll a, b, total chlorophyll, and carotenoid content were determined after the incubation of fresh leaves (0.2 g each replicate) with 10 mL of 100% methanol for 24 h at 4 °C. Methanolic extract was determined by reading the absorbance at 665 nm, 652 nm, and 470 nm in a SHIMADZU UV-1800 spectrophotometer. The pigment contents were determined using the proper formulas reported by Lichtenthaler [33].

Fresh leaves (0.2 g) were homogenized with 2 mL of 70% aqueous methanol, kept 30 min in ice, and centrifuged at 14,000× *g* for 20 min. The supernatant was used for the biochemical determinations of total polyphenol content (TPC) and antioxidant activity. TPC was determined using a modified protocol of the Folin Ciocalteau method [34]. The analysis was performed in triplicate using 10 μL of supernatant. The incubation was performed at 40 °C for 30 min, then the absorbance was spectrophotometrically determined at 765 nm. TPC was expressed as mg of GAE per g of FW (mg gallic acid equivalents per g of fresh weight). 

#### 4.4.2. Antioxidant Activity

Antioxidant activity was determined using the DPPH (2,2-diphenyl-1-picrylhydrazyl radical) and ABTS (2,2-azino-bis(ethylbenzenethiazoline-6-sulfonic acid) assays. In the DPPH assay, aliquot of 10 μL of supernatant was used for the spectrophotometric determination at 517 nm of the blenching of DPPH after 30 min of incubation at room temperature in the dark [35]. Trolox was used as the control (2.5 mM), and the activity was referred as µmol Trolox Eq/g FW.

The ABTS assay was assessed in triplicate according to the method proposed by Re et al. [36], by using 10 μL of supernatant. ABTS radical was generated by 10 μL of supernatant with 7.0 mM ABTS-solution and 2.45 mM potassium persulfate solution. After exactly 5 min, the absorbance of the reaction mixture was measured at 734 nm. Trolox was used as the control (2.5 mM). The results were expressed as mM of Trolox equivalents per 1 g of fresh leaves. 

### 4.5. Phytochemical Analyses

#### 4.5.1. Non-Volatile Compounds Analysis

##### Extract Preparation

The aerial parts of three *S. rebaudiana* samples were first dried and then subjected to extraction in a Soxhlet apparatus using methanol as the solvent in a ratio 1:10 g/mL. The solvent was successively removed under vacuum to obtain the three final extracts that were used for phytochemical analyses. The yields of the extraction process are shown in Table 5.

##### High-Performance Liquid Chromatography–Mass Spectrometry (HPLC–MS)

The chemical profiles of the *S. rebaudiana* extracts were performed using a high-performance liquid chromatography coupled to a photodiode array detector and a mass spectrometer (HPLC-PDA-ESI-MS). The system was composed of a Surveyor LC pump, a Surveyor autosampler, a Surveyor PDA detector, and a LCQ Advantage ion trap mass spectrometer (Thermo Finnigan, San Jose, CA, USA) equipped with an electrospray ionization source. The analyses were performed on a Synergi Fusion-RP column of 4.6 × 250 mm with 4 µm particle size (Phenomenex, Milan, Italy) eluting with a mixture of methanol (solvent A) and a 0.1% aqueous solution of formic acid (solvent B) using a solvent gradient 5–95% A in 90 min. The samples were dissolved in methanol (2.5 mg/mL), centrifuged, and injected into the LC–MS system (injection volume 20 μL). The elution was performed at a flow rate of 0.8 mL/min with a splitting system of 2:8 to MS detector (160 µL/min) and PDA detector (640 µL/min), respectively. The analyses were performed with an ESI interface in both negative and positive ionization modes, with a scan range of *m*/*z* 150–2000. The ESI-MS/MS conditions were optimized as previously reported (Tavarini et al., 2021): spray voltage, 4.50 kV; capillary temperature, 270 °C; capillary voltage, −16.0 V negative mode and +21.0 V positive mode; tube lens offset, −5 V; sheath gas flow rate, 60.00 arbitrary units; auxiliary gas flow rate, 3.00 arbitrary units; and normalized collision energy 35%. N^2^ was used as the sheath and auxiliary gas. The PDA data were recorded with 200–600 nm range. The MS data were elaborated using Xcalibur 3.1 software.

The amount of steviol glycosides in all the extracts was obtained using rebaudioside A as an external standard in a concentration range of 125–1000 µg/mL. Solutions of rebaudioside A standard at five different concentrations were prepared and injected in triplicate into the LC–PDA/MS system. The MS data were recorded in SIM (single ion monitoring) mode, useful for the accurate quantification of analytes. The areas obtained from the integration of the peaks were used to obtain the calibration curve, and a linear simple correlation was used to investigate the relation between variables (*R*^2^ 0.9632). The amount of steviol glycosides was expressed as mg/g of dry weight (DW).

#### 4.5.2. Volatile Compound Analysis

##### Headspace-Solid Phase Microextraction (HS-SPME) Analysis

The spontaneous emission of volatiles was analyzed using the headspace-solid phase microextraction (HS-SPME) technique. Each sample (5 leaves each) was placed individually into a 50 mL glass flask, then covered with aluminum foil and allowed to equilibrate for 30 min at room temperature. A Supelco SPME (Solid Phase Micro-Extraction) device coated with polydimethylsiloxane (PDMS, 100 μm), previously preconditioned according to the manufacturer’s instructions, was used to sample the headspace. The sampling was accomplished in an air-conditioned room (22 ± 1 °C) to guarantee a stable temperature. After the equilibration time, the fiber was exposed to the headspace for 30 min. Both the equilibration and sampling times were experimentally determined to obtain an optimal adsorption of the volatiles, and to avoid both under- and over-saturation of the fiber and of the mass spectrometer ion trap. Once the sampling was finished, the fiber was withdrawn into a needle and immediately transferred to the injection port of the GC–MS system. The desorption conditions were identical for all the samples (indicated in Section 2.3). Furthermore, blanks were performed before each first SPME extraction and randomly repeated during each series. 

##### Gas Chromatography–Mass Spectrometry (GC–MS)

The GC/EI–MS analyses were performed using a Varian CP-3800 apparatus equipped with a DB-5 capillary column (30 m × 0.25 mm i.d., film thickness 0.25 μm) and a Varian Saturn 2000 ion-trap mass detector. The oven temperature was programmed to rise from 60 to 240 °C at 3 °C/min; the injector temperature was set at 220 °C; the transfer-line temperature was set at 240 °C; and the carrier gas (He) flow was set at 1 mL/min. The acquisition parameters were the following: full scan with scan range: 35–300 *m/z*; scan time: 1.0 s; and threshold: 1 count. The identification of the constituents was based on a comparison of their retention times (*t_R_*) with those of pure reference samples and their linear retention indices (LRIs) determined relatively to the *t_R_* of a series of *n*-alkanes. The mass spectra were compared to those listed in the commercial libraries NIST 14 and ADAMS, a home-made mass-spectral library built up from pure substances and components of known oils, and the MS literature data [37,38,39,40,41]. 

## 5. Conclusions

Continuing the investigation on the adaptation of different plant species to underwater conditions, the present work studied *S. rebaudiana* cultivated both in pot and in a hydroponic system. The obtained results evidenced a modification in the plant metabolism. In fact, besides the differences in the volatile chemical composition, the most important change, as observed by both micromorphological and biochemical analyses, was a reduction of polyphenol content from the plants grown in the underwater environment and, consequently, their lower antioxidant activity. Moreover, a reduction of steviol glycosides was also evidenced between the control and the Nemo’s potted sample, while the Nemo’s hydroponic sample showed a content of these molecules comparable to the terrestrial plant. This aspect makes the Nemo’s Garden^®^ hydroponic system a good alternative to the traditional agricultural system for the cultivation of *S. rebaudiana*, as this species has gained increasing attention in recent times for its ability to synthesize steviol glycoside. Furthermore, its marked chlorophyll a/b ratio, similar to that of the control sample, is indicative of a good quality for both plant cultivation and photosynthetic apparatus.

## Figures and Tables

**Table 1:** Determination of metabolites in *Stevia rebaudiana* leaves collected from the Nemo’s Garden^®^ and from the control plants grown in terrestrial aerial condition. The data are presented as means ± standard error (SE, *n* = 3). Abbreviations: DW = dry weight; GAE—gallic acid equivalents; TE = Trolox equivalent.

	Nemo’s Hydroponic	Nemo’s Pot	Control Pot
Chlorophylla (chl a) mg/g FW	0.69 ± 0.15	0.47 ± 0.02	0.71 ± 0.1
Chlorophyll b (chl b) mg/g FW	0.36 ± 0.05	0.34 ± 0.01	0.37 ± 0.05
Total Chlorophylls (Tchl) mg/g FW	1.05 ± 0.05	0.81 ± 0.03	1.09 ± 0.15
Ratio chl a/chl b	2.1	1.3	1.9
Total Carotenoids (TCar) mg/g DW	0.23 ± 0.02	0.05 ± 0.01	0.13 ± 0.02
Total phenolics (TPC) mg GAE/g DW	15.83 ± 2.39	10.55 ± 0.76	26.6 ± 0.44
Antioxidant DPPH-assay mmol TE /mL	3.78 ± 1.03	1.77 ± 0.8	7.4 ± 1.66
Antioxidant ABTS activity mmol TE /mL	3.00 ± 0.36	1.91 ± 0.4	4.34 ± 0.46

**Table 2:** Chromatographic (*t_R_*, retention time), UV (λ_max_, wavelength of maximunm absorbance), and full/fragmentation mass spectrometry data (MS and MS/MS) of compounds detected in the methanolic extracts of *Stevia rebaudiana* using HPLC-PDA-ESI-MS analyses. The peak numbers correspond to those of Figure 3.

Peak	Compound ^a^	*t_R_* (min)	[M-H]^−^	[M+HCOO]^−^	[M+Na]^+^	MS/MS Ions (*m*/*z*)	λ_max_	Extracts
**1**	Dicaffeoylquinic acid I	38.2	515		-	**353**, 179	252, 328	CP, NH, NP
**2**	Dicaffeoylquinic acid II	38.8	515		-	**353**, 179	249, 329	CP, NH, NP
**3**	Dicaffeoylquinic acid III	43.1	515		-	**353**, 179	248, 330	CP, NH, NP
**4**	Quercetin rhamnoside	44.9	447		-	**301**, 179, 151	257, 346	CP, NH, NP
**5**	Dicaffeoylquinic acid III	45.5	515		-	**353**, 179	255, 331	CP, NH, NP
**6**	Quercetin rhamnosyl-diglucoside	46.9	771		-	**609**, 301	257, 335	CP, NH, NP
**7**	Tricaffeoylquinic acid	48.1	677		-	**515**, 353	257, 332	CP, NH, NP
**8**	Rebaudioside A	52.3	965	1011	989	827, **665** ^b^	-	CP, NH, NP
**9**	Rebaudioside D	54.0	1127	1173	1151	989, **827** ^b^	-	CP, NH, NP
**10**	Rebaudioside N	55.1	1273	1319	1297	-	-	CP, NH, NP
**11**	Rebaudioside F	56.2	935	981	959	-	-	CP, NH, NP
**12**	Stevioside	60.2	803	849	827	**665**, 509 ^b^	-	CP, NH, NP
**13**	Rebaudioside C	61.9	949	995	973	**811**, 665 ^b^	-	CP, NH, NP
**14**	Rebaudioside B	62.3	803	849	827	**665**, 503 ^b^	-	CP, NH, NP

^a^ Tentatively identified based on UV, ESI-MS, and ESI-MS/MS data; ^a^ the base ion peak is shown in bold. ^b^ Product ions generated by the fragmentation of the parent ion [M+Na]^+^. CP = Control Pot; NH = Nemo’s Hydroponic; NP = Nemo’s Pot.

**Table 3:** Main steviol glycosides in the three analyzed samples of *Stevia rebaudiana.* The results are reported as means ± standard deviation (mg/g of dried aerial parts ± SD, *n* = 3).

Peak	Compound	Nemo’s Hydroponic	Nemo’s Pot	Control Pot
**12**	Stevioside	43.3 ± 17	25.1 ± 1.8	44.3 ± 6.7
**13**	Rebaudioside C	1.46 ± 0.26	0.793 ± 0.048	0.743 ± 0.15
Total		44.8 ± 17	25.9 ± 1.8	45.0 ± 6.8

**Table 4:** Complete chemical composition of the headspace of the three analyzed samples of *Stevia rebaudiana*. The data are presented as relative abundance (%) ± standard deviation (SD, *n* = 3).

Compounds	l.r.i. ^1^	Class	Relative Abundance (%)		
			Nemo’s Hydroponic	Nemo’s Pot	Control Pot
α-pinene	933	MH	- ^2^	-	0.6 ± 0.10
sabinene	976	MH	-	-	1.7 ± 0.18
β-pinene	982	MH	1.5 ± 0.06	1.3 ± 0.05	9.1 ± 0.25
2-octanone	992	NT	-	-	-
*(E)-*3-hexenol acetate	1002	NT	-	0.2 ± 0.00	-
*(Z)-*3-hexenol acetate	1009	NT	-	-	-
limonene	1029	MH	-	-	0.7 ± 0.04
1,8-cineole	1032	OM		-	0.2 ± 0.05
*(E)-*β-ocimene	1052	MH	0.4 ± 0.02	-	
linalool	1101	OM	0.4 ± 0.01	0.2 ± 0.01	0.5 ± 0.11
nonanal	1104	NT	-	-	0.4 ± 0.11
*(E)-*4,8-dimethylnona-1,3,7-triene	1116	NT	-	-	-
*(Z)-*3-hexenyl butyrate	1186	NT	-	-	-
α-terpineol	1189	OM	0.1 ± 0.01	-	0.2 ± 0.00
decanal	1204	NT	-	-	0.5 ± 0.01
*(Z)-*3-hexenyl isovalerate	1234	NT	-	-	-
*(E)-*hex-3-enyl *(E)-*2-methylbut-2-enoate	1319	NT	-	-	-
δ-elemene	1340	SH	8.0 ± 0.07	6.1 ± 0.18	1.5 ± 0.01
cyclosativene	1368	SH	0.1 ± 0.01	-	-
α-copaene	1376	SH	0.3 ± 0.01	0.3 ± 0.04	-
β-bourbonene	1384	SH	-	0.3 ± 0.00	-
β-cubebene	1390	SH	0.4 ± 0.02	-	0.9 ± 0.01
β-elemene	1392	SH	2.5 ± 0.01	2.4 ± 0.01	2.1 ± 0.01
*(Z)-*caryophyllene	1405	SH	-	0.1 ± 0.01	-
α-gurjunene	1410	SH	0.4 ± 0.03	0.4 ± 0.03	0.5 ± 0.01
β-ylangene	1414	SH	-	2.8 ± 0.19	-
β-caryophyllene	1420	SH	21.1 ± 0.45	27.5 ± 0.26	36.0 ± 0.19
β-copaene	1429	SH	-	-	0.6 ± 0.01
γ-elemene	1433	SH	2.2 ± 0.12	-	-
*trans*-α-bergamotene	1438	SH	1.7 ± 0.12	1.9 ± 0.09	2.5 ± 0.01
aromadendrene	1445	SH	0.2 ± 0.07	0.2 ± 0.07	-
α-humulene	1456	SH	7.9 ± 0.07	8.8 ± 0.01	6.7 ± 0.04
*(E)-*β-farnesene	1460	SH	10.4 ± 0.04	10.0 ± 0.59	8.1 ± 0.12
*allo*aromadendrene	1461	SH	-	-	-
*cis*-muurola-4(14),5-diene	1462	SH	-	-	-
γ-muurolene	1477	SH	0.4 ± 0.12	0.3 ± 0.17	-
germacrene D	1478	SH	10.9 ± 0.01	10.9 ± 0.46	13.1 ± 0.07
*ar*-curcumene	1483	SH	-	-	-
β-chamigrene	1485	SH	0.1 ± 0.08	0.6 ± 0.21	-
valencene	1492	SH	-	0.5 ± 0.40	-
bicyclogermacrene	1496	SH	19.5 ± 0.09	14.8 ± 0.04	5.2 ± 0.03
δ-amorphene	1505	SH	-	0.2 ± 0.02	-
α-bulnesene	1505	SH	2.4 ± 0.10	1.9 ± 0.01	-
β-bisabolene	1509	SH	0.8 ± 0.04	0.9 ± 0.02	-
*trans*-γ-cadinene	1513	SH	1.2 ± 0.04	1.3 ± 0.01	1.3 ± 0.01
δ-cadinene	1525	SH	1.3 ± 0.01	1.4 ± 0.02	1.6 ± 0.01
*(E)-*γ-bisabolene	1535	SH	0.3 ± 0.03	0.3 ± 0.00	-
α-cadinene	1538	SH	0.2 ± 0.04	0.2 ± 0.00	-
*(E)-*nerolidol	1565	OS	0.3 ± 0.05	0.2 ± 0.02	0.7 ± 0.00
germacrene D-4-ol	1575	OS	1.2 ± 0.03	0.7 ± 0.02	0.4 ± 0.01
caryophyllene oxide	1581	OS	0.4 ± 0.03	0.7 ± 0.01	-
globulol	1583	OS	0.2 ± 0.00	-	-
5-*epi*-7-epi-α-eudesmol	1603	OS	0.2 ± 0.05	-	-
humulene epoxide II	1608	OS	-	0.1 ± 0.06	-
γ-eudesmol	1630	OS	0.2 ± 0.03	-	-
*epi*-α-cadinol	1641	OS	0.4 ± 0.04	0.4 ± 0.00	1.1 ± 0.10
manoyl oxide	1988	OD	1.8 ± 0.00	0.9 ± 0.02	3.8 ± 0.02
Monoterpene hydrocarbons (MHs)			1.9 ± 0.08	1.3 ± 0.05	12.1 ± 0.37
Oxygenated monoterpenes (OMs)			0.5 ± 0.00	0.2 ± 0.01	0.9 ± 0.16
Sesquiterpene hydrocarbons (SHs)			92.1 ± 0.16	93.9 ± 0.23	80.1 ± 0.51
Oxygenated sesquiterpenes (OSs)			2.7 ± 0.21	2.1 ± 0.11	2.2 ± 0.10
Oxygenated diterpenes (ODs)			1.8 ± 0.00	0.9 ± 0.02	3.8 ± 0.02
Non-terpene derivatives (NTs)			-	0.2 ± 0.00	0.9 ± 0.10
Total identified (%)			98.92 ± 0.02	98.6 ± 0.4	100.0 ± 0.01

^1^ Linear retention indices on a DB5 capillary column. ^2^ Not detected.

**Table 5:** Yields of the extraction process of the three analyzed samples of *Stevia rebaudiana*.

	Nemo’s Hydroponic	Nemo’s Pot	Control Pot
Plant dry weight (g)	5.71	2.97	24.5
Methanol extract (g)	2.81	0.97	9.66
Yield (%)	49.3	32.5	39.4

## Data Availability

Not applicable.

## Sample Availability

Not available.
